# Healthcare professionals' perceptions of the acceptability of the PREVENT‐PE trial: A mixed‐methods survey and interview study

**DOI:** 10.1111/aogs.70249

**Published:** 2026-05-26

**Authors:** Angel Leung, James Goadsby, Kayleigh S. Sheen, Sergio A. Silverio, Laura A. Magee, Peter von Dadelszen, Argyro Syngelaki, Kypros H. Nicolaides

**Affiliations:** ^1^ Harris Birthright Research Centre for Fetal Medicine King's College Hospital London UK; ^2^ Department of Women & Children's Health, School of Life Course & Population Health Sciences King's College London London UK; ^3^ Department of Social Sciences, College of Health, Science and Society University of the West of England Bristol UK; ^4^ Department of Psychology, Institute of Population Health University of Liverpool Liverpool UK

**Keywords:** healthcare professionals, pre‐eclampsia acceptability, staff surveys, trial evaluation

## Abstract

**Introduction:**

There is a lack of published data on how healthcare professionals perceive the acceptability of third‐trimester risk screening in pregnancy. The PREVENT‐PE trial [ISRCTN41632964] demonstrated that a personalized approach to term pre‐eclampsia risk assessment to inform risk‐stratified timed birth at term reduced the incidence of term PE by 30%, without increasing Cesarean birth or neonatal morbidity. We aimed to evaluate the acceptability of the PREVENT‐PE trial among healthcare professionals.

**Material and Methods:**

Staff were invited to an online survey comprising validated metrics of acceptability, which were analyzed descriptively. Also, the staff were invited to one‐to‐one interviews to probe the acceptability of the trial in more depth. Using template analysis, responses were mapped onto the following domains of acceptability: Sense‐making (Intervention Coherence), Commitment (Affective Attitude), Change (Burden), Communication (Ethicality), Motivation (Opportunity Costs), Action (Self‐Efficacy), and Evaluation (Perceived Effectiveness).

**Results:**

There were 65 completed surveys, whilst 19 healthcare professionals participated in interviews. Forty‐one staff participants were from King's College Hospital, and twenty‐one staff participants were from Medway Maritime Hospital, in line with the recruitment of participants to the trial. Approximately 29% of staff were of non‐White ethnicity. Most staff were doctors or midwives, in similar proportions. One‐third of staff participants had no prior involvement in clinical trials. Acceptability was endorsed by the majority of survey respondents (69.2%), as “very acceptable” (44.6%) or “somewhat acceptable” (24.6%). Interview findings endorsed acceptability from most staff toward participating in an evidence‐based trial such as PREVENT‐PE. Despite some concerns about implementation burden, system‐level readiness, and counseling high‐risk participants, the staff supported integrating such screening into routine maternity care if effectiveness is confirmed.

**Conclusions:**

The PREVENT‐PE trial had a high level of acceptability among healthcare professionals. Staff view risk screening in the third trimester of pregnancy as acceptable, with potential to positively impact patient outcomes.

## Graphical abstract

The entire study was informed by an established acceptability framework for healthcare interventions, which has seven domains: Intervention Coherence, Affective Attitude, Burden, Ethicality, Opportunity Costs, Self‐Efficacy, and Perceived Effectiveness. These domains have been descriptively extended to factor in the human services element of each aspect, ensuring that while the implementation elements of acceptability are prominent, the way in which they have been interpreted and experienced can also be understood.

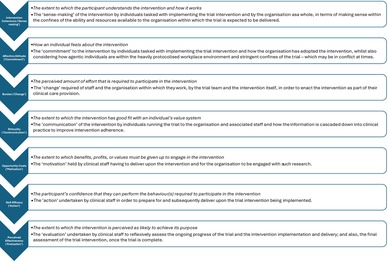

**Abbreviations**
FMF: Fetal Medicine Foundation
NHS: National Health Service
PE: pre‐eclampsia
PREVENT‐PE: The Prevention of Pre‐eclampsia Trial

**Key message**

Screening for pre‐eclampsia risk at 35–36 weeks' gestation and offering risk‐stratified, planned early term birth was both acceptable and achievable. Staff felt motivated by the clinical rationale for the trial and the potential benefits for patients.

## 1 INTRODUCTION

Pre‐eclampsia (PE) is a major global cause of maternal and neonatal morbidity and mortality, complicating up to 5% of all pregnancies.^1^ PE occurs most commonly at term, when it contributes to the majority of maternal complications and a third of PE‐associated newborn complications.^2^ Whilst this makes the term PE of major public health interest, there was no intervention that reduced its incidence. Aspirin from early pregnancy did not reduce term PE, nor did pravastatin from 36 weeks' gestation.^3^, ^4^ However, timed birth before 40 weeks' gestation was shown to improve clinical outcomes in low‐risk nulliparous women,^5^ making timed birth an intervention of interest, particularly as it is one that can be offered in all health systems.

The PREVENT‐PE trial aimed to test whether a strategy of screening for PE risk at 35–36 weeks' gestation and offering risk‐stratified, planned early term birth could reduce the incidence of subsequent PE, without adversely affecting rates of emergency Cesarean birth or neonatal morbidity. “PREVENT‐PE” was an open, randomized trial of 8,136 unselected women who were attending a hospital for a routine fetal ultrasound scan. Following consent, women were randomized to the intervention (PE risk assessment by the Fetal Medicine Foundation [FMF] competing risks model algorithm at 35^+0^ to 36^+6^ weeks' gestation, and for risk ≥1‐in‐50, risk‐stratified planned early term birth) or control (usual care at term). The intervention (vs. control) was associated with a 30% reduction in the incidence of the primary outcome of PE, with no difference in emergency Cesarean or neonatal unit admission ≥48 h.^6^

To support the implementation of clinical trial results, clinicians and policy‐makers require information beyond clinical outcomes. These include experiences of receiving care (by participants) and providing that care (by staff) in the context of the trial. Patient uptake in PREVENT‐PE was high (75%), adherence to the intervention was good (83%), and few participants withdrew (0.1%), suggesting the intervention was acceptable to women.^6^, ^7^ However, these data provide no insight into staff experiences, such as clinical trial management and understanding of randomization procedures, understanding of potential benefits (such as improved patient outcomes), and reassurance about evidence‐based practice.^8^ There is a lack of available data on the attitudes of midwives and obstetricians regarding the use of iatrogenic delivery for the reduction of clinical risk, and generally a lack of data on attitudes to methods of timed birth (i.e., labor induction or elective Cesarean), beyond studies of induction protocols focusing on the complex logistics of delivery. Yet, staff experiences are key drivers of not only staff involvement and research success, including recruitment and retention of participants, fidelity to the protocol, familiarity with the intervention, and patient safety, but also enhanced validity of the trial data and their generalizability and implementation.^9^, ^10^, ^11^, ^12^ In particular, it is important for those implementing trial interventions to be aware of challenges experienced in the trial, such as stakeholder collaboration, communication gaps, and regulatory hurdles.^8^

Through a structured survey and in‐depth interviews, this study sought to assess PREVENT‐PE trial acceptability among healthcare professionals who worked in recruitment to, or delivery of, PREVENT‐PE. The ultimate goal was to determine the feasibility of implementing a timed birth protocol to reduce term PE incidence. These data add further perspective to the ongoing debate around the role of labor induction and Cesarean birth in modern, risk‐conscious obstetric practice.

## 2 MATERIAL AND METHODS

### 2.1 Ethical approval and research governance

This paper reports the views of staff who were recruited to, or worked on delivering, the PREVENT‐PE trial, from May 9, 2023, until June 7, 2024, at King's College Hospital National Health Service (NHS) Foundation Trust, London, and Medway NHS Foundation Trust, Gillingham, UK. The protocol^13^ and results^6^ have been published and were summarized above.

### 2.2 The novel PREVENT‐PE intervention

The intervention was a strategy of PE risk assessment using the FMF competing‐risks model, and then risk‐stratified timed birth.

The externally validated FMF competing‐risks model combines maternal factors with biomarkers to estimate individual patient‐specific risks of PE,^6^ Box 1. The model has been externally validated and is available online, embedded in FMF software as an app for routine clinical use or accessible directly through the website for one‐off, individual case assessments by clinicians or researchers.

BOX 1 **FMF Competing‐risks model.**

**Table:** 

Maternal factors
Maternal age (years)
Body weight (kg)
Height (cm)
Ethnic group
Method of conception
Chronic hypertension
Pre‐existing diabetes
Systemic lupus erythematosus
Antiphospholipid syndrome
Parity, including previous pregnancy with PE
Interpregnancy interval (years)
Family history of PE
Biomarkers
Mean arterial blood pressure (mmHg)
Serum concentrations of placental growth factor (MoM)
Soluble FMS‐like tyrosine kinase‐1 (MoM)
*N/B*. Variables measured continuously.
MoM, multiples of the median.

For risk‐stratified timed birth in the intervention group, participants with a PE risk of at least one in 50 were offered risk‐stratified planned early term birth at 37, 38, 39, or 40 weeks' gestation. Participants with higher PE risk were offered birth earlier than those with a lower risk.^6^, ^13^ Initiation of birth was by labor induction (by local protocol) or elective cesarean (if indicated or desired by the woman) within the first 2 days of the gestational week of planned birth. Participants with a risk of PE below one in 50 were considered to be at low risk of PE and received usual care, as in the control group.

At both study sites, fetal ultrasound is routinely performed, but not PE risk assessment at 35–36 weeks, or the novel offer of risk‐stratified timed birth. The success of the trial required changes in the way that risk at term was approached and managed. Healthcare professionals had to be educated about the term PE risk screening methodology and the implications of risks so generated, to better support patients who were offered timed birth at a given gestational age. Also, staff were required to facilitate timed birth, such as through administrative scheduling, undertaking labor induction, or delivering intrapartum care, including performing elective Cesareans by maternal choice (rather than labor induction), when at high risk of term PE. These necessary steps may have generated resistance to the implementation of the trial protocol. There was potential for a lack of understanding of how the protocol could work to reduce term PE rates, a view that implementation may lead to additional workload or that timed deliveries may be performed “unnecessarily.”

### 2.3 Data collection

As outlined in the protocol,^13^ PREVENT‐PE included an implementation‐evaluation component to supplement data on clinical outcomes. A variety of evaluation approaches were utilized, including staff and patient surveys and interviews. This study focuses on the acceptability of the intervention amongst healthcare professionals, with patient data reported elsewhere.^7^

The online staff survey was hosted by Qualtrics and was open for completion by healthcare professionals from March 6 to October 22, 2024. Relevant maternity services staff were recruited from King's College Hospital NHS Foundation Trust, Medway NHS Foundation Trust, and the FMF. Maternity care staff were e‐mailed *en masse* to participate in the survey, if appropriate and interested; also, they were asked to share their contact details if they were willing to have an individual interview. Each survey took approximately 3–5 min to complete, and the interviews were scheduled for 30 min.

The entire study was informed by an established acceptability framework for healthcare interventions.^14^ See Box 2 for full explanations of each of the framework's seven domains. These domains have been descriptively extended to factor in the human services element of each aspect, ensuring that whilst the implementation elements of acceptability are prominent, the way in which they have been interpreted and experienced can also be understood.

Survey questions to assess staff acceptability of the PREVENT‐PE trial were adapted from guidance on developing questionnaires from an established framework for acceptability of healthcare interventions.^15^ Items from Sekhon's Theory Informed Questionnaire were tailored to refer to the PREVENT‐PE trial and screening for PE specifically. Response options were presented on a 5‐point Likert scale, with higher scores representing a more positive endorsement of the item (see Appendix S1 for the survey). Items were shaped by theory, existing empirical evidence, and stakeholder perspectives, with a key goal for facilitating assessment of acceptability for any trial.^15^

Raw survey and interview data were handled by independent evaluators [KSS, SAS] and were not accessible to the core PREVENT‐PE team. Descriptive analyses were undertaken for all study variables using SPSS (version 30).

Questions for the semi‐structured interview schedule were developed using the same acceptability framework as for the survey.^14^ Interviews were conducted on a one‐to‐one basis, and began with participants being asked about their role and relationship to the PREVENT‐PE Trial, then participants were asked questions which addressed and reflected each domain of acceptability in Box 2. Please see Appendix S2 for the interview schedule.

BOX 2 **Acceptability framework domains (adapted from Sekhon et al., 2017)^14^**

Intervention coherence (“Sense making”): The extent to which the participant understands the intervention and how it works. When coherence is thought to be high, staff are more likely to see it as logical and compatible with their workload model and the value system of their work and workplace.

Affective Attitude (“Commitment”): How an individual feels about the intervention. Those who have positive evaluations of the trial and intervention to foster commitment and willingness to engage consistently over time to meet delivery targets and ensure safe and ethical recruitment.

Burden (“Change”): The perceived amount of effort that is required to participate in the intervention. Communication must therefore be effective to ensure an alignment between personal and organizational values held, and those introduced by the trial intervention, while the main focus must be on building trust in the intervention.

Ethicality (“Communication”): The extent to which the intervention has a good fit with an individual's value system. Participants are more likely to engage with and endorse interventions they perceive as respectful, fair, and culturally appropriate.

Opportunity Costs (“Motivation”): The extent to which benefits, profits, or values must be given up to engage in the intervention. Where losses are perceived as greater or riskier or where benefits are not seen to be immediately beneficial, motivation can be weakened, leading to delay in recruitment or lack of adherence.

Self‐Efficacy (“Action”): The participant's confidence that they can perform the behavior(s) required to participate in the intervention. Those with higher levels of self‐efficacy can promote collective action by increasing persistence and supporting others when difficulties occur.

Perceived Effectiveness (“Evaluation”): The extent to which the intervention is perceived as likely to achieve its purpose. Evaluative perception in clinical effectiveness may contribute to the reinforcement of organizational motivation to engage in such research and encourage sustained participation by individual clinical staff, who will often evaluate a trial's effectiveness based on early experiences, progression of the trial, feedback, and troubleshooting; positive reinforcement and reward for recruitment targets being met; dissemination of findings, and observed outcomes.

### 2.4 Data analyses

This was a descriptive qualitative analysis whereby qualitative data from interviews were mapped onto specific acceptability domains (Box 2), using template analysis.^16^ These domains were utilized as a commonly‐employed acceptability check for implementation‐evaluation studies, and our test was to see whether these domains were fulfilled or if there was refutation from the interviewed participants about the different aspects of acceptability. The analysis was therefore confirmatory in nature, and did not set out to be experientially‐focused or exploratory. The absence of data for any one domain of acceptability would have signaled a lack of acceptability from the staff's perspective; the presence of data for each domain signaling endorsement.

Template analysis was chosen for its pragmatic approach to coding based on previously‐defined concepts—in this case, an implementation science acceptability framework.^17^ To map data onto domains of acceptability allowed us to assess whether the implementation of the trial intervention had satisfied thresholds of acceptability for staff who were expected to deliver on the trial intervention. Template analysis relies on a methodical six‐step process of: re‐familiarization with the data (i.e., re‐reading transcripts); preliminary coding (i.e., broadly categorizing the data into the template's themes); organization of themes into the template (i.e., moving themes around to produce a more logical narrative order of themes); defining the template (i.e., providing definitions for each of the themes); application of the final template to the full dataset (i.e., completing the coding of all transcripts); and finalization of template definitions (i.e., agreeing a finalized set of themes and associated definitions in the template as a whole). Coding and analysis are done iteratively and reflexively (with the researcher reflecting on their own role in the process), and analysts check for accuracy of coding and interpretation throughout.

## 3 RESULTS

### 3.1 Demographics

Complete survey responses were received from 65 staff members, of whom 19 also underwent interviews.

The characteristics of survey respondents are presented in Table 1. Two‐thirds of staff participants were from King's College Hospital, in‐line with the recruitment of participants to the trial. Approximately 20% of staff were of non‐White ethnicity. Most staff were doctors or midwives, in similar proportions. One‐third of staff participants had no prior involvement in clinical trials; their involvement in PREVENT‐PE was clinical in almost half, and both clinical and research in another 27%. The vast majority of staff had contact with potential PREVENT‐PE patients at least weekly.

**TABLE 1:** Characteristics of survey respondents.

Trust site	*N*	%
King's College Hospital NHS Foundation Trust	41	63.10
Medway Maritime Hospital NHS Foundation Trust	23	35.40
Prefer not to say and missing	1	1.50
Ethnicity		
White British	29	44.60
White Other	23	35.40
Black	2	3.10
Asian	4	6.10
Mixed	4	6.10
Prefer not to say and missing	3	4.60
Professional role		
Consultant, obstetric consultant (with/without gynecology)	10	15.40
Doctor	6	9.20
Clinical Research Fellow	13	20.00
Sonographer	2	3.10
Midwife	32	49.20
Maternity care assistant	1	1.50
Prefer not to say and missing	1	1.50
Previous involvement in clinical trials		
Yes	45	69.20
No	20	30.80
Capacity of involvement with the PREVENT‐Trial		
Clinical	29	44.60
Research	11	16.90
Both	18	27.70
Indirectly involved	7	10.80
Previously involvement in clinical trials		
Yes	45	69.20
No	20	30.80
Frequency of contact with patients in relation to the PREVENT Trial		
Daily	8	12.30
4–6 times per week	7	10.80
2–3 times per week	20	30.80
Once per week	17	26.20
Never	4	6.20
Other	9	13.80

### 3.2 Survey analysis

Table 2 shows that of the 65 survey respondents, the majority (69.2%) found PREVENT‐PE to be acceptable, with 44.6% scoring it as “very acceptable” and 24.6% as “somewhat acceptable.” A minority of staff (16.9%) considered the trial to be unacceptable, primarily “somewhat unacceptable” (10.8%), rather than “very unacceptable” (6.2%); 13.8% of staff remained neutral.

**TABLE 2:** Participant views on acceptability (frequency of endorsement for each item, split by allocation group).

Survey question	Response (*N*, %)				
	Very positive	Positive	Neutral	Somewhat negative	Negative
How acceptable was the PREVENT trial to you?	29 (44.60%)	16 (24.60%)	9 (13.80%)	7 (10.80%)	4 (6.20%)
How fair is the PREVENT Trial for people at risk of pre‐eclampsia?	32 (49.20%)	14 (21.50%)	13 (20.00%)	4 (6.20%)	2 (3.10%)
How comfortable did you feel being part of the PREVENT trial?	13 (20%)	14 (21.50%)	10 (25.40%)	11 (16.90%)	17 (26.20%)
It is clear to me how timed birth will help to improve outcomes after screening for pre‐eclampsia at 35–36 weeks' gestation	25 (38.50%)	18 (27.70%)	11 (16.90%)	8 (12.30%)	3 (4.60%)
How confident did you feel about engaging with the PREVENT trial (i.e., did you have the information that you needed)?	27 (41.50%)	19 (29.20%)	12 (18.50%)	4 (6.20%)	3 (4.60%)
How confident did you feel in answering questions from participants about the PREVENT trial?	18 (27.70%)	25 (38.50%)	10 (15.40%)	9 (13.80%)	3 (4.60%)
How easy was it to discuss the PREVENT trial with participants, providing information about low risk?	25 (38.50%)	18 (27.70%)	13 (20.00%)	8 (12.30%)	1 (1.50%)
How easy was it to discuss the PREVENT trial with participants, providing information about high risk?	17 (26.20%)	24 (36.90%)	16 (24.60%)	7 (10.80%)	1 (1.50%)
How much effort did it take to be involved in the PREVENT Trial?	23 (35.40%)	19 (27.70%)	14 (21.50%)	9 (13.80%)	1 (1.50%)
Being involved in the PREVENT trial interfered with my other priorities.	9 (12.30%)	12 (18.50%)	20 (30.80%)	7 (10.80%)	18 (27.70%)
The PREVENT Trial has improved birth outcomes	20 (30.80%)	9 (13.80%)	27 (41.50%)	5 (7.70%)	4 (6.20%)

Across the different questions, most staff found the trial fair and easy to discuss with participants, especially with a low‐risk case (66.2%) (Table 2). However, a notable proportion of staff had concerns. Despite most (66.2%) staff being clear on how timed birth would help to improve outcomes, some staff found communication with high‐risk cases challenging (63.1%), which aligned with the just under half (41.5%), who expressed “comfort in participation” (20% “very comfortable” and 21.5% “somewhat comfortable”), and the 43.1% who felt uncomfortable (either “very uncomfortable” [26.2%] or “somewhat uncomfortable” [16.9%]). Nevertheless, a distinct minority (15.3%) of staff expressed that the trial would require significant effort, and a minority (30.8%) felt the trial would interfere with other priorities. Almost half (44.6%) of staff believed that the trial had improved birth outcomes, with a similar proportion (41.5%) having a neutral view.

Overall, the results suggest that the PREVENT‐PE trial was generally well‐received and accepted by staff, but that counseling of women regarding risk‐stratified timed birth presented some challenges.

### 3.3 Interview analysis

Analysis of the 19 staff interviews is presented below, according to the domains of acceptability used.^14^ Although we utilized this established acceptability framework as the basis of our template, we extended our description to capture the human services element of each domain. Below, each theme (based on each domain of acceptability) is presented, with a short description of the domain as per the original framework and our extension of its meaning. Associated illustrative quotations are presented in Tables 3 and 4.

**TABLE 3:** Quotations from template themes (1‐3), mapped to acceptability domains.

Theme	Illustrative quotations
(1) Intervention Coherence (sense making)	It's a valid research question to be asked. I think it might change the care of women. It could find something interesting. (Participant 8)
	When I first heard about the trial, I had reservations on how it would like pragmatically to translate into clinical practise just because on the face of it, it does seem like something that would increase the clinical workload in terms of increasing intervention through early through inductions or electives c‐sections, but actually. Once I sort of looked into all the rationale behind it and realised that actually, you know, you're largely talking about cohort of women who probably would be induced a week or so later. (Participant 9)
	With the members of research group was really useful is that we when evolved into a more sophisticated discussion thinking about hierarchies of risk and therefore taking one group will be 37 weeks, one group will be 38, one 39, one 40 and the others offer an induction of 41 weeks… we will reduce the burden of still birth or we won't be able to measure it. Because you're reducing high risk, women earlier, so therefore they can't be stillbirth (Participant 13)
	I'm absolutely convinced that that was a very needed study that needed to be carried out and that was based on evolution of all of the research that has been published through the Fetal Medicine Research Institute and the Fetal Medicine Foundation over the last several years and decades. That demonstrate that it is feasible to screen for preeclampsia here that is Objective 1 to say that can it be effectively? Can you effectively identify a proportion of women who are likely to develop the condition ahead of them developing it? And 2nd is that having identified the women at potential risk, is there a management plan that can actually prevent them from developing preeclampsia here and the whole idea of preventing preeclampsia here was Effective screening at 36 weeks identify a cohort of women. What extremely high risk and offer them time to delivery and then to calculate the cohort of women who developed preeclampsia and those who don't, based on the way they are randomised, to see if timely delivery can prevent the incidence of preeclampsia term without a potential increase in the risk of any adverse outcomes. (Participant16)
(2) Affective attitude (commitment)	It's been a difficult balance as a midwife to, you know, midwives kind of traditionally more sort of non‐interventionists. once I got into it, it's. It's just making sure that. Everybody was aware of what their screening was and was aware of how accurate their screening was, and everyone was informed, and then everybody also knows the risks about, you know, early intervention, whether that's induction or c‐sections and all of that. So actually, I didn't feel that being committed to the trial was any sort of conflict of interest. As also being a clinical midwife.(Participant 9)
	I think allowing people to take time to consider their screening and to take time to consider the recommendations is key… Pushing them or rushing them, then that trust is much harder to build. (Participant 9)
	Having a bit more time prior to opening the trial would have been really beneficial because it was it felt as much as we. We knew about the trial. We knew it's in the pipeline. It wasn't confirmed until about 2 weeks before we opened it. It felt like there was not enough time. It felt they rushed which ultimately I don't feel impacted the trial too much, but I would say we would have benefited from having a little bit more time with being with staff training outside of research and fetal medicine because we have got over 200 midwives, so as much as we did make every effort to train all of the midwife, having a bit more time to do that before we started the trial would be really beneficial rather than us sort of training as we've already opened the trial, I think that would have been really helpful. (Participant 12)
	All of the staff who were not about the study team, they're on board. So I think one of the key things is to have a phase in period to allow wider dissemination of the study objectives, allow all people within an infrastructure whether they're a part of the study team or not a part of the study team to be able to reflect on the study protocol, the implications of the study protocol. The ability to have questions and be answered and address those questions in a constructive way really was very helpful for us. (Participant 14)
(3) Burden (change)	You must incorporate individualised care in maternity and that is exactly the point of the prevent trial you personalise the care of a woman based on her characteristics, her the results of her blood pressure, of the blood and you give a specific risk and based on that risk you do a recommendation (Participant 1)
	It would be hard to convince women to be part of this project or that lots of women would not want to and that was something I was concerned about, that women just would not want to be part of a project where they may need to be induced or delivered like any earlier than they would need to be (Participant 2)
	I can say with confidence that there is very little money. There isn't enough money to do to do what's on the agenda now and so. I think we need to be the pragmatic one and this isn't an expensive intervention. (Participant 4)
	I think they feel completely overwhelmed because they are coming here for the last scan and it's a lot of information together with four consent forms, four information sheets. So, it's a lot and you don't give enough time to read because you don't have time. (Participant 6)
	It's those kind of logistical challenges, whereas with our other studies we have a bit more flexibility in terms of when we've been patients in to see them, you can kind of sit around meetings and things like that, whereas prevent you can't fit it around with anything. You have to drop what you're doing if there's a prevent patient and then if they are randomised to screen (Participant 12)

**TABLE 4:** Quotations from template themes (4‐7), mapped to acceptability domains.

Theme	Illustrative quotations
(4) Ethicality (communication)	This study resonates with the women and the staff. I think the larger problem would be in the implementation with policy makers…. What we're doing and see how we offer the most evidence informed care that we can and you have those conversations, and of course, just because you offer a time birth does not mean that the woman either wants it or accept it but that's her choice (Participant 4)
	There has been a lot of ongoing discussions and actually what I used to do in the sort of earlier days was anybody that screened high risk that wanted to talk to their community midwife. The community midwife didn't know about the trial and we would then sort of join in their appointments and have like three‐way conversation about it and think that helps to sort of spread the information as well. (Participant 9)
	I think the challenge is more sort of the time and having access to people to have those conversations. Once we had the conversations, I feel like everyone that I spoke to in the end had a much better understanding and much more committed on board with the trial. (Participant 12)
	Within the research team, the feedback has been overwhelmingly positive. The only questions or some criticism or some questions rather than criticism we've had are with the clinical teams on the delivery suite because the arm of women that we're going to be randomised, we're going to have a delivery at this particular place and time. And one of the concerns was we have not had major issues with because through effective discussions and team meetings we have been able to explain that the number of women who would be eligible to do that would be far would be quite small. (Participant 16)
(5) Opportunity costs (Motivation)	I want an answer and I want an answer that I can take all the way through to implementation… All important and very often when clinical trials are undertaken, when there is no difference in the clinical outcomes and they're cash strapped at the end. They spend the money for the health economic analysis, and they only do that as important if the intervention is effective, there's an actual fact if the outcomes are similar and one and the intervention is actually cheaper (Participant 4)
	No one wants to see women being unwell from developing something in pregnancy. So that is definitely kind of a big motivation of keeping going. At times it has been tough. I think because of a lot of the staff kind of pushing back on it, that has been challenging. (Participant 9)
	Hopefully we'll see that actually delivering these women and has made a big impact rather than sitting on them for longer periods of time, and then they do become preeclamptic at a later stage (Participant 11)
	If that is proven, we would definitely change our local policies and guidelines to say that to keep it consistent with latest evidence based research, we would give women that information shared that information with stakeholders and the lay groups and the support groups to change your policies and pathways to implement this in clinical practise, if it was proven that this works. (Participant16)
(6) Self‐Efficacy (Action)	So, here is again another thing to fight. You have sonographers, you have clinics, you have midwives, you is another every single and other several components that you have to convince that what you're doing is right. It's not enough just to publish papers and say this is a useful thing to do. (Participant 1)
	I always relied on things that could control. So, I was uncertain how things would work out. So that was the main thing everything for the last 40 years, all my research I controlled completely and here was something that goes beyond me, I depend on the goodwill or competence of others (Participant 3)
	The research midwives did a very good job being the hybrid in between the patients who had concern and how to deal with the labour ward, they did a very good job as in being the middle ground. Obviously, the staff downstairs counselled the patients to the point that they have no questions, and they will just go into delivery… it was quite nice trial to see that everyone collaborating together without fighting. (Participant 6)
	I felt from experiencing in PREVENT that… actually we've got a long way to go and getting our midwives to trust research before we can get our participants to trust research because they are gatekeepers in maternity. (Participant 14)
(7) Perceived effectiveness (Evaluation)	We are quite effective in preventing preterm preeclampsia but they have no impact on term preeclampsia, so we knew that. We were not good in predicting or preventing term preeclampsia, and yet we looked at the outcome data and we found that the contribution of term preeclampsia, overall maternal and perinatal adverse events was quite important (Participant 3)
	I don't understand why there's so much barrier sometimes for the NHS and national guidance to implement recent evidence but thrives in so far it is usually a quite a big delay. I don't have an answer to that. (Participant 8)
	So a positive study here if it proves that you could actually prevent preeclampsia by screening all potential women identifying a high risk group and actually preventing the complication has major implications for clinical practise nationally within the UK, but also internationally. (Participant 16)
	I'm familiar with the system and I know that takes ages to implement any positive results expected any possibilities to that will reach public and at the hospitals and the prenatal be implemented and results of the trial with proves that it's useful. (Participant 17)

#### 3.3.1 Intervention coherence

In our data, most staff reported a high level of understanding of the PREVENT‐PE intervention. They discussed how the goal of prevention of term PE was a valid endeavor, and that the intervention was evidence‐based and had been designed to address this goal. Whilst some participants shared their reservations about the nature of the intervention, citing fundamental differences in how interventional birth is viewed by the professional disciplines of obstetrics and midwifery, it was emphasized that the acceptance of the trial would depend ultimately on its effectiveness.

#### 3.3.2 Affective attitude

The findings from our study suggested that staff generally demonstrated a high level of commitment toward research and the trial specifically. This was enhanced by updates on recruitment progress, which for this trial was for more than 8000 women. With such a large trial, some participants discussed how it would be helpful to have more time for preparation, with two components receiving particular emphasis. First, to enhance the commitment of all staff involved in the trial, more time was recommended for staff training, particularly in a cross‐disciplinary setting, where women are cared for in hospital and in the community, and many staff who are involved with trial participants are outside of the core research team. Second, more time was recommended for the development of the research culture, particularly outside the hospital setting, where community midwives were providing post‐randomization antenatal care visits from 35^+0^ to 36^+6^ weeks' gestation and birth.

#### 3.3.3 Burden

From the findings of our study, staff were happy to adapt to the changes required for trial delivery due to their belief in the rationale for the trial. However, some staff did express concern about whether the current constraints on services would make it possible for the NHS to implement the results of trials such as PREVENT‐PE.

#### 3.3.4 Ethicality

In our data, most staff reported that at the beginning of the trial, there was some miscommunication between research staff, community midwives, and trial participants. However, this improved over time, following further training and ongoing discussion; as such, staff generally felt the trial was aligned with their clinical competencies and that it could be delivered with fidelity to the protocol.

#### 3.3.5 Opportunity costs

All staff interviewed were highly motivated to contribute to finding an answer to the study's over‐arching research question, to reduce the burden of term PE. Staff believed that the trial should be implemented globally if the results were successful, as long as hospital infrastructure could accommodate it.

#### 3.3.6 Self‐efficacy

From the results of this study, staff were confident about their ability to play their role(s) within the research teams, although some highlighted challenges faced initially with community midwives outside the Trust, whose support had not yet been secured when randomization had started.

#### 3.3.7 Perceived effectiveness

Our study results demonstrated that staff were all very willing to implement practice change if the trial results achieved more benefit than harm. However, all staff expressed concern about how the NHS would implement the intervention nationally and the length of time it would take to roll out the intervention across all Trusts.

## 4 DISCUSSION

This mixed‐methods evaluation of staff's perspectives of PREVENT‐PE trial delivery provides important insights into the acceptability of implementing a third‐trimester PE risk screening and early birth strategy to prevent term PE. Most staff surveyed (69.2%) found the trial to be acceptable, with strong support for the trial's rationale. Interview analysis further reinforced these findings, revealing staff endorsement of intervention coherence, affective commitment, perceived effectiveness, and, in particular, acceptability. Some concerns were expressed about implementation burden and system‐level readiness, and communication challenges (particularly between staff and community midwives); however, staff supported integrating such screening into routine maternity care if effectiveness were confirmed.

Well‐trained staff can offer personalized support and have a positive impact on prospective participants' willingness to participate, initially and throughout the trial. Important elements of discussion include: the study's rationale; the nature, potential benefits, and potential risks of the intervention; and the burden and frequency of visits. Understanding these factors improves recruitment and reduces protocol deviations, each of which improves the generalizability and validity of trial results.^9^, ^10^ The high (75.0%) recruitment rate among potential participants mirrored our staff acceptability findings. This, and the high retention within the trial (97.7%), suggests that staff's endorsement may facilitate participants' trust and engagement, supported by previous literature linking staff enthusiasm with recruitment efficacy.^10^, ^12^ The ability of staff to communicate effectively with participants, especially around those patients with a high‐risk result, was found to be both a challenge and a critical component of implementation devotion. Furthermore, the previous findings^18^ reveal a persistent underreporting of evidence‐based benefits in NHS labor induction information leaflets, where most leaflets compared induction with spontaneous (rather than expectantly‐managed) labor (which may result in either spontaneous or iatrogenic birth). Such miscommunication and under‐reporting may undermine staff's efforts in counseling and bias women's decision to participate despite effective communication from staff.

This study lays the groundwork for evidence‐based implementation of the PREVENT‐PE intervention into clinical practice, through strong alignment between staff belief in the trial's rationale, willingness to engage in its delivery, and the ability to reconcile professional values with research commitments. Trial staff's acceptance of such a novel intervention should give clinical staff confidence to incorporate this new approach into their daily practice,^11^, ^12^ as well as supporting the sustainability of change. Previous research^9^, ^11^ has shown successful trials are strengthened by clinician engagement, particularly in interventions that modify established care pathways, as is the case with the PREVENT‐PE trial.^6^

Nevertheless, there was a clash evident in our findings between the different views of midwives and obstetricians who view pregnancy and childbirth as physiological or potentially pathological, respectively.^19^, ^20^ This may result in midwifery professionals being more willing to undertake watchful waiting for the onset of spontaneous labor, whereas obstetricians may be more ready to intervene with planned time birth. Nevertheless, both professional groups support informed, shared decision‐making and personalized care, and each is informed by the PREVENT‐PE findings.

This study has several strengths, including the use of a validated theoretical framework for acceptability,^15^ a mixed‐methods design, and inclusion of diverse perspectives from staff in both clinical and research roles. However, there are limitations to consider. Most staff were from the same NHS Foundation trust, which could impact the generalizability of our findings. Additionally, the under‐representation of community midwives, who play a critical role in maternity care, may have limited insights into barriers within the eight domains of acceptability. Furthermore, deductive structuring of analysis can be criticized for its reductionist focus; however, with such an implementation‐evaluation study as we present here, our interest is narrow—on the acceptability of the trial intervention, and we are not attempting to provide any experiential insight into the lived experience of being involved as a member of staff in a clinical trial.

Future research should consider: involving more NHS Foundation trust sites; examining any changes in staff acceptability throughout the trial and following knowledge of trial results; and continuing to capture patients' and community stakeholders' perspectives. Particular attention should be paid to midwifery and obstetric perspectives. Focused implementation planning may ensure that interventions like PREVENT‐PE are integrated into routine practice, without placing undue pressure on NHS services.

## 5 CONCLUSION

This mixed‐method evaluation of staff views within PREVENT‐PE found that a strategy of screening for PE risk at 35–36 weeks' gestation and offering risk‐stratified, planned early term birth was both acceptable and achievable. Staff felt motivated by the clinical rationale for the trial and the potential benefits for patients. The logistical and structural issues that arose, particularly early in the trial, were related to communication, workload, and system capacity. They emphasize the need to engage staff in designing and implementing complex interventions for successful study delivery and post‐study implementation.

## AUTHOR CONTRIBUTIONS

AL: coordinated data collection, participated in interpretation of the data, accessed and verified the underlying data reported in the manuscript, and wrote the first draft of the manuscript. JG: acquired the necessary funding, coordinated data collection, and participated in the interpretation of data. KSS: informed methods of data collection, analyzed the survey data, and participated in the interpretation of data. SAS: informed methods of data collection, analyzed the qualitative data, participated in the interpretation of all data, and assisted in writing the first draft of the manuscript. LAM: conceived and designed the study, participated in the interpretation of all data, and assisted in writing the first draft of the manuscript. PvD: conceived and designed the study and participated in the interpretation of data. AS: conceived and designed the study, supervised the conduct of the study, participated in the interpretation of data, and accessed and verified the underlying data reported in the manuscript. KHN: conceived and designed the study, supervised the conduct of the study, and participated in the interpretation of data. All authors edited the manuscript and approved the final version for submission.

## FUNDING INFORMATION

The study was supported by a grant from the Fetal Medicine Foundation (UK Charity No: 1037116). Reagents and equipment for the measurement of serum PlGF and sFlt‐1 were provided free of charge by Thermo Fisher Scientific.

## CONFLICT OF INTEREST STATEMENT

All authors declare that they have no known competing financial interests or personal relationships that could have influenced the work reported. All authors confirm that they had full access to all the data in the study and accept responsibility to submit for publication.

## ETHICS STATEMENT

The results of the PREVENT‐PE Trial (prospectively registered: ISRCTN41632964) have been published. The trial was approved by the National Health Service Health Research Authority, London—Surrey Research Ethics Committee (reference 22/LO/0794, 5 Apr 2023), and all study participants provided written, informed consent. The trial was registered (ISRCTN41632964, 1 Nov 2022) prior to recruitment.

## Supporting information


**Appendix S1:** Staff acceptability survey.
**Appendix S2**. Staff interview schedule.

## Data Availability

The datasets generated and/or analyzed during this study are not publicly available due to their sensitive and potentially identifiable nature. However, a de‐identified dataset may be available from the corresponding author, upon reasonable request, with input from the co‐investigator group where applicable, and in accordance with the data sharing policies of King's College Hospital.
