# Supplementary material for: Healthcare professionals' perceptions of the acceptability of the PREVENT‐PE trial: A mixed‐methods survey and interview study
Source: Acta Obstet Gynecol Scand. 2026 May 26;105(8):1542–52. doi: 10.1111/aogs.70249 (PMC13356467; doi:10.1111/aogs.70249)
Supplement: Supplementary file 1 — Appendix S1: Staff acceptability survey. Appendix S2. Staff interview schedule. [file AOGS-105-1542-s001.docx]

**SUPPLEMENTARY APPENDIX**

| **Item** | **Title** | **Page number** |
| --- | --- | --- |
| S1 | Staff acceptability survey | 2-3 |
| S2 | Staff interview schedule | 4-5 |

**Appendix 1**

**Staff acceptability survey**

**PREVENT Trial – Quantitative – Staff survey**

**Section One** will record participant characteristics, information about their current professional role, and information on their involvement in PREVENT. This will facilitate description of participants, comparison of representativeness, and may provide further context about any answers provided

**Section Two** is an adapted version of Sekhon’s Theory-Informed Questionnaire (2022) to assess the acceptability of healthcare interventions. It addresses each domain relevant for addressing acceptability. Items have been adapted to specify involvement in the PREVENT trial. Additional items have been inserted to address perspectives on discussing the trial with participants and answering questions. These have been inserted to reflect elements important for women receiving communication about high-risk obstetric circumstances (Hilder et al. 2020).

**Section Three** is a single item that will serve to identify those willing to provide further feedback about the trial in an interview

| **SECTION ONE: PARTICIPANT CHARACTERISTICS** | | |
| --- | --- | --- |
| Demographics | 1. | Age |
|  | 2. | Gender |
|  | 3. | Ethnicity |
| Professional role | 4. | Current professional role |
|  | 5. | Length of time in current role |
|  | 6. | Which hospital Trust |
|  | 7. | Length of time at Trust |
|  | 8. | Capacity with regards to PREVENT (Clinical/research/both) |
|  | 9. | Previous involvement in clinical trials (yes/no) |
| PREVENT trial involvement | 10. | Length of time involved in PREVENT trial |
|  | 11. | Capacity within which involved in PREVENT trial |
|  | 12. | Approximate frequency of contact with potential/actual participants |

| **SECTION TWO: ASSESSMENT OF ACCEPTABILITY (ADAPTED FROM SEKHON ET AL. 2022)** | | | |
| --- | --- | --- | --- |
| Affective attitude | 1. | How comfortable did you feel being involved with the PREVENT trial? | 1 Very uncomfortable – 5 Very comfortable |
| Burden | 2. | How much effort did it take to engage with the PREVENT trial? | 1 No effort at all – 5 Huge effort |
| Ethicality | 3. | How fair is the PREVENT trial for people ~~with signs~~ at risk of pre-eclampsia? | 1 Very unfair – 5 Very fair |
| Perceived effectiveness | 4. | The PREVENT trial has improved birth outcomes | 1 Strongly disagree – 5 Strongly agree |
| Intervention coherence | 5. | It is clear to me how timed birth will help to improve outcomes after screening for pre-eclampsia at 35-36 weeks’ gestation | 1 Strongly disagree -5 Strongly agree |
| Self-efficacy | 6. | How confident did you feel about engaging with the PREVENT trial (i.e., did you have the information that you needed)? | 1 Very unconfident – 5 Very confident |
| Adapted for this study | 7. | How easy was it to discuss the PREVENT trial with participants?  - information about low risk?  - information about high risk? | 1 Not easy at all – 5 Very easy |
|  | 8. | How confident did you feel in answering questions from participants about the PREVENT trial? | 1 Very unconfident – 5 Very confident |
|  | 9. | Were there any elements that you found harder to talk about? | [free text] |
| Opportunity costs | 10. | Being involved in the PREVENT trial interfered with my other priorities | 1 Strongly disagree -5 Strongly agree |
| General | 11. | How acceptable was the PREVENT trial to you? | 1 Not acceptable at all- 5 Very acceptable |
| Adapted for this study | 12. | What could have been improved about the PREVENT trial? | [free text] |
|  | 13. | What are some of the barriers to timed birth for pregnancies at increased risk of pre-eclampsia? | [free text] |
|  | 14. | Can you think of any ways in which screening for risk could be improved? | [free text] |
|  | 15. | Do you have any other feedback or comments? | [free text] |

| **SECTION THREE: INVITATION TO INTERVIEW** | | | |
| --- | --- | --- | --- |
| Link to qualitative follow up | 1. | Would you be willing to provide further information about your perspectives on the PREVENT trial as part of a brief interview?  If so, please provide your contact details below and a member of the research team will be in touch with further information |  |
|  |  | Name: |  |
|  |  | Email address: |  |
|  |  | Telephone number: |  |
|  |  | Alternative telephone number: |  |
|  |  | Preferred time to contact: (AM, PM, early evening): |  |

**Thank you so much for your time!**

**References**

Hilder, J., Stubbe, M., Macdonald, L., Abels, P., & Dowell, A. C. (2020). Communication in high risk ante-natal consultations: a direct observational study of interactions between patients and obstetricians. *BMC Pregnancy and Childbirth*, *20*(1), 1-13.

Sekhon, M., Cartwright, M., & Francis, J. J. (2022). Development of a theory-informed questionnaire to assess the acceptability of healthcare interventions. *BMC Health Services Research*, *22*(1), 279.

**Appendix 2**

**Staff interview schedule**

**PREVENT Trial – Qualitative – Staff Interview Schedule.**

Thank you for taking the time to be interviewed in relation to The PREVENT Trial. My name is <Name of Researcher> from <Name of Institution> and I am part of an evaluation team who have been commissioned to undertake this evaluation. You have had the opportunity to consider the Participant Information Sheet and sign the Consent Form. The interview will take approximately 30 minutes and your identity will be kept completely anonymous. Do you have any questions before we begin? If you are happy, I will now start to record.

**Role:** *May I start with asking some questions about your role.*

- What is your current role? What organisation and client group do you work with?
- Do you have clinical responsibilities, research responsibilities, or both?
- What has been your involvement with The PREVENT Trial to date?

**Sense-making (Intervention Coherence):** *I would now like now to ask some questions about The PREVENT Trial itself as I would like to get a better idea of your sense of The PREVENT Trial.*

- Could you tell me your understanding of the aims of The PREVENT Trial?
- Could you to tell me about your initial reactions to The PREVENT Trial?
  - Any comparisons/how fits with other clinical practice
  - Understanding of its core components
- Overall, do you feel The PREVENT Trial will be useful in clinical practice?

**Commitment (Affective Attitude):** *I would now like to talk about commitment in relation to The PREVENT Trial both from an individual perspective and an organisational perspective.*

- What are your thoughts in relation to your own accountability in delivering on The PREVENT Trial?
  - Commitment of self and colleagues to The PREVENT Trial?
  - How might The PREVENT Trial be better established with frontline staff?

**Change (Burden):** *Please can we move on now to thinking about aspects of change to clinical practice and The PREVENT Trial*

- How does The PREVENT Trial fit in with other changes going on in your organisation?
  - Experience of previous change in your organisation/colleagues’ views

**Communication (Ethicality):** *A further area I would like you to think about now is communication in relation to The PREVENT Trial*

- How far have you been able to discuss or engage others in The PREVENT Trial and ‘spread the word’?
  - Specific examples of dissemination?
- What feedback, if any, have you received on The PREVENT Trial?
  - Any discussions with your colleagues? In what context? If not, why not?
  - How do you think your colleagues feel about it?
  - Have there been any differences in opinion between colleagues in your organisation? How have these been resolved?

**Motivation (Opportunity Costs):** *Moving on from communication I would like to undertake a bit of a costs-benefits analysis with you.*

- What are your expectations about The PREVENT Trial? What are you going to do with it?
- What has motivated you to date, to engage with The PREVENT Trial?
- Any perceived opportunity costs for engaging in and/or implementing The PREVENT Trial?
- Can you think of anything that might better facilitate moving forward with the engaging with The PREVENT Trial?
  - Training? Resources? Support?

**Action (Self-Efficacy):** *Linking to your appraisal of the costs-benefits analysis we have just conducted I would now like us to focus on taking things forward from an individual and organisational perspective*

- Are there any enablers to engaging with The PREVENT Trial for you individually?
- Are there any enablers to engaging with The PREVENT Trial organisationally?
- Are there any barriers to engaging with The PREVENT Trial for you individually?
- Are there any barriers to engaging with The PREVENT Trial organisationally?

**Evaluation (Perceived Effectiveness):** *Finally, could we discuss any potential evaluation you have made of The PREVENT Trial to date.*

- Do you think The PREVENT Trial will make a difference to your patients?
- Do you think The PREVENT Trial will make a difference to clinical practice, both your own and in the wider healthcare professional community?
- Do you think The PREVENT Trial will make a difference to your organisation?
  - *For all of the above:* If so, why? If not, why not?

**End of interview:** *My final questions are more around advice sharing.*

- Do you have any advice for The PREVENT Trial team or those engaged with the Trial?
- Any advice for other clinicians like yourself wanting to be involved in The PREVENT Trial or research more broadly?
- Any advice for women who may be approached about taking part in The PREVENT Trial or any other research during pregnancy and childbirth?
- I have come to the end of my questions, but is there anything you would like to add which I might not have asked you about?
- Do you have any questions for me?
- Are you happy for what you have said today to be used in our analysis?

Thank you for your time, I’m going to turn the tape off and then we can have a quick debrief before I close the call.
